# Modernizing the Toolkit for Arthropod Bloodmeal Identification

**DOI:** 10.3390/insects12010037

**Published:** 2021-01-06

**Authors:** Erin M. Borland, Rebekah C. Kading

**Affiliations:** Department of Microbiology, Immunology, and Pathology, Colorado State University, Fort Collins, CO 80523, USA; rebekah.kading@colostate.edu

**Keywords:** arthropod vectors, bloodmeal analysis, molecular barcoding, molecular advances, field-forward

## Abstract

**Simple Summary:**

The ability to identify the source of vertebrate blood in mosquitoes, ticks, and other blood-feeding arthropod vectors greatly enhances our knowledge of how vector-borne pathogens are spread. The source of the bloodmeal is identified by analyzing the remnants of blood remaining in the arthropod at the time of capture, though this is often fraught with challenges. This review provides a roadmap and guide for those considering modern techniques for arthropod bloodmeal identification with a focus on progress made in the field over the past decade. We highlight genome regions that can be used to identify the vertebrate source of arthropod bloodmeals as well as technological advances made in other fields that have introduced innovative new ways to identify vertebrate meal source based on unique properties of the DNA sequence, protein signatures, or residual molecules present in the blood. Additionally, engineering progress in miniaturization has led to a number of field-deployable technologies that bring the laboratory directly to the arthropods at the site of collection. Although many of these advancements have helped to address the technical challenges of the past, the challenge of successfully analyzing degraded DNA in bloodmeals remains to be solved.

**Abstract:**

Understanding vertebrate–vector interactions is vitally important for understanding the transmission dynamics of arthropod-vectored pathogens and depends on the ability to accurately identify the vertebrate source of blood-engorged arthropods in field collections using molecular methods. A decade ago, molecular techniques being applied to arthropod blood meal identification were thoroughly reviewed, but there have been significant advancements in the techniques and technologies available since that time. This review highlights the available diagnostic markers in mitochondrial and nuclear DNA and discusses their benefits and shortcomings for use in molecular identification assays. Advances in real-time PCR, high resolution melting analysis, digital PCR, next generation sequencing, microsphere assays, mass spectrometry, and stable isotope analysis each offer novel approaches and advantages to bloodmeal analysis that have gained traction in the field. New, field-forward technologies and platforms have also come into use that offer promising solutions for point-of-care and remote field deployment for rapid bloodmeal source identification. Some of the lessons learned over the last decade, particularly in the fields of DNA barcoding and sequence analysis, are discussed. Though many advancements have been made, technical challenges remain concerning the prevention of sample degradation both by the arthropod before the sample has been obtained and during storage. This review provides a roadmap and guide for those considering modern techniques for arthropod bloodmeal identification and reviews how advances in molecular technology over the past decade have been applied in this unique biomedical context.

## 1. Introduction

Approximately a quarter of pathogen outbreaks affecting humans in the 20th century were spread by hematophagous arthropod vectors, showcasing the important role of vector surveillance, ecological research, and vector control in public health practice [1]. Vertebrate–vector interactions drive transmission and establishment of vector-borne pathogens, but many aspects of this complex web still remain to be elucidated. Molecular identification of arthropod bloodmeal sources has become an increasingly important tool as the global public health community seeks to further understand the transmission dynamics of arthropod-vectored pathogens [2]. Laboratory methods for identifying the tiny bloodmeals of engorged arthropods have evolved substantially over the decades, while the epidemiological significance bloodmeal identification brings to research has remained critical and constant.

Early techniques for bloodmeal identification, such as precipitin tests developed early in the 20th century and enzyme-linked immunosorbent assays (ELISA) popular in the 1980s, were limited in their ability to identify vertebrate hosts to only those species for which anti-sera had been generated. These tests also had low species-specificity, restricting identification to order, family, or genus [3,4]. The adoption of molecular techniques beginning in the 1990s, including DNA sequencing, allowed researchers to break these barriers and reach into genus- and species-level identifications, in some cases making the identification of individual people possible.

A decade ago, Kent [2] reviewed the available methods for bloodmeal identification, yet many changes and improvements have come into vogue in the field since then. New technologies have been introduced, including quantitative PCR (qPCR) and next generation sequencing (NGS) protocols, which have come into widespread use as associated costs have decreased. This review highlights molecular advancements in bloodmeal identification made over the past decade since previously reviewed [2], and briefly discusses traditional methods that remain useful for cost effective analysis.

## 2. Diagnostic Markers: Mitochondrial DNA

**Figure 1:**
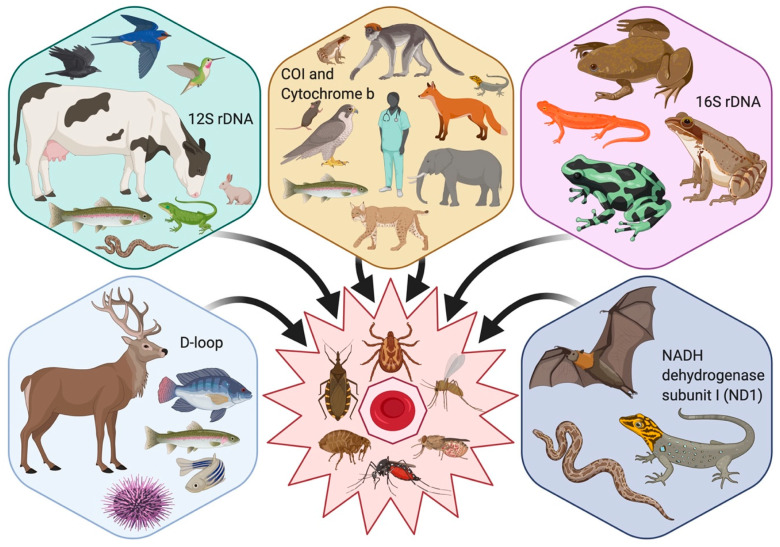
A wide variety of animals have been identified using mitochondrial DNA (mtDNA) molecular markers for bloodmeal analysis. A representative subset associated with each marker is shown here [9,10,11,12,13,14,15,16,17,18,19,20,21,22,23,24,25,26,27,28,29,30,31,32]. This figure was created using BioRender.com.

Mitochondrial DNA (mtDNA) remains a popular and advantageous sequencing target for arthropod bloodmeal identification [2]. Mitochondria are universal to eukaryotic organisms and the 13 protein-encoding genes across the approximately 16.6 kilobases of closed-circular mtDNA are easily amplified using available technologies [5]. The economy of organization of the mtDNA, particularly rarity of insertions and deletions (indels) and the lack of introns due to its prokaryotic origins, is beneficial for comparisons across orders or families where differences in nuclear genome composition and organization can pose challenges for sequence alignment [5]. High copy numbers of clonal mtDNA per cell allow successful identification of microscopic traces of animal materials without the need for cloning, which reinforces the application of mtDNA assays to the bloodmeals of small hematophagous arthropods (i.e., mosquitoes) where the quantity of blood is miniscule [6,7]. In nearly all eukaryotes, mtDNA is maternally inherited and recombination is extremely rare [8]; the result being that all changes to the sequence are due solely to mutation which allows a clean evolutionary history to be established. As a result of the features of mtDNA, studies across all blood feeding arthropods in the last decade have utilized mtDNA as the molecular target of interest (Figure 1).

### 2.1. Cytochrome c Oxidase Subunit I (COI)

In 2003, Hebert et al. first proposed a 658 nucleotide fragment encompassing the 5′ end of the COI gene called the “Folmer Region” for species barcoding [33,34]. In this context, a “barcode” represents a specific DNA sequence associated with a particular taxonomic group, and it has been demonstrated that unique COI profiles could be used to identify and discriminate organisms to the species level [9]. The rationale for choosing COI as a barcoding locus for cataloguing biodiversity was that the gene provides robust coverage of many species and possesses a higher rate of mutation, thought to be roughly three times that of 12S or 16S rDNA [35]. COI has the added benefit that it is not prone to the indels that plague other genes, which makes alignment across diverse taxa more straightforward [36]. Utilization of COI as a molecular target is facilitated by its adoption by the Barcode of Life Consortium as their barcoding region of choice and availability of reference barcodes for 227,000 animal species in the Barcode of Life Database (BOLD) as of 2020 [33,37]. COI has the greatest reference database representation of any molecular target, with the possible exception of Cyt b, making it an attractive and useful target.

In addition to the longer Folmer Region, other sub-regions of COI have been used in pursuit of molecular barcoding. Short fragment sequences roughly 350 nucleotides in length have been applied to the analysis of the semi-digested predatory fish gut contents with success [38] as well as the blood meals of parasitic isopods in aquatic systems [31]. In an effort to assist in sequencing heavily degraded DNA, 100 to 300 nucleotide mini-barcodes were designed for use on museum specimens and other samples preserved in DNA-degrading conditions [39]. Mini-barcoding has been applied to samples that were dried or cooked, fixed in formalin, or found in permafrost and other harsh conditions, so it is likely that mini-barcoding could easily be applied to identification of semi-degraded bloodmeals. In addition, clever design of multiple overlapping mini-barcodes may be used to sequence the entirety of the COI locus should greater species resolution be required [40,41].

### 2.2. Cytochrome b (Cyt b)

In 1989, Kocher et al. [11] first targeted Cyt b, which was originally used for species delimitation by taxonomists and for forensic purposes. It was not long before the region was adopted for determining the source of arthropod bloodmeals, and Cyt b remains widely used either alone or in tandem with other loci. When compared to COI, Cyt b has greater variation despite the sequence being shorter, has similar intraspecific variation to COI, exhibits a lower rate of false positives, and has a greater positive predictor value [12]. In addition, Cyt b-based phylogenies better match taxonomy-based phylogenetic trees across taxa [12]. In addition, the portion of the gene that is typically targeted is around 400 nucleotides, and the small fragment size is beneficial for use with degraded or trace samples [42]. Amplification of a small, 98 nucleotide section of Cyt b was paired with restriction enzyme digest to identify the hosts of mosquito bloodmeals for up to 60 h after feeding, making this target useful for identification of digested bloodmeals [43].

### 2.3. 12S and 16S Mitochondrial Ribosomal DNA (rDNA)

Both 12S and 16S rDNA genes have been utilized for species identification with variable results. The alignment of 12S and 16S sequences are complicated by the presence of indels in many species, as well as extreme sequence length polymorphisms [21,33]. This makes comparison across diverse species more complicated, but offers the opportunity to compare length differences as a potential diagnostic marker [35]. The mutation rate of 16S is roughly three times slower than that of protein coding genes including COI and Cyt b, and both 12S and 16S exhibit a 3′ end that is largely invariant in many species [35,44]. Despite this, mutations are common in a few variable regions which may be sufficient to molecularly distinguish many vertebrates to the species level, but success has been far from universal [45].

Bloodmeal sources have been identified by 12S barcoding and mini-barcoding including mammals, birds, and reptiles, and these assays remain popular in the identification of the meals of ticks and *Triatominae* [13,14,15,16,17,18]. For fish, a hypervariable region of the 12S gene is a common target in conjunction with COI and Cyt b [19,20]. While for some arthropods fish are not a common meal source, some mosquito species are known to feed on air breathing or amphibious fish in swamp environments [32].

For amphibians, 16S is suggested as a secondary barcoding locus due to high variability in the conserved region of COI that has led to difficulty in primer design and gene amplification for many amphibian species [21]. The lower mutation rate of 16S can eliminate these challenges, though sometimes this can result in reduced resolution power due to overlap of interspecific and intraspecific divergences [46]. The reduced diversity of the gene also can preclude the speciation of samples, with resolution only to genus or higher taxa [47]. This locus may be of use to those studying the feeding habits of *Corethrellidae*, a family of biting midges known to specialize on frogs and reptiles, as well as *Ixodes pacificus* ticks, the nymphal stage of which is a lizard specialist [48,49].

One focus of 12S and 16S barcoding has been for the recovery of heavily degraded DNA from traditional medicinal and dried or cooked food products. These loci show potential for application to the barcoding of partially digested bloodmeals in which the DNA of the vertebrate host has been similarly degraded. The development of a 12S “mini-barcode” for the identification of degraded fish products shows potential for use identifying heavily degraded bloodmeals of arthropods [50]. Similarly, a mini-barcode targeting approximately 200 nucleotides near the 3′ end of the 16S gene was successfully used to identify heavily processed samples and meat mixtures [45]. Despite the small fragment size, a large portion of the diversity of 16S comes from indels, making this critical information in the separation of samples that can be harnessed for speciation [45,51]. Mini-barcoding could be useful to identify bloodmeals from previous life stages of some arthropods, such as ticks, which digest blood sources intracellularly and maintain trace amounts through ecdysis [52,53].

### 2.4. NADH Dehydrogenase Subunit I (ND1)

To counter loci-associated drawbacks for some taxonomic groups, additional mtDNA regions have been explored for greater species resolution. The ND1 gene has been estimated to have better sequence variability than COI [54], and has been employed successfully for taxonomic studies of bats [22,23], reptiles [24,25], and amphibians [26,27]. While reptiles and amphibians are not typically the hosts of arthropod feeding, identifying a marker that can successfully speciate these animals is of importance since some mosquito species, including those in the genus *Uranotaenia*, take the majority of their meals from ectotherms [55,56]. When used in conjunction with other loci such as COI or Cyt b, this region has increased resolving power.

### 2.5. D-Loop

One of the newest loci to be targeted is the mtDNA d-loop, which is a part of the non-coding region of the mtDNA and is incorporated as a third strand of DNA approximately 650 nucleotides in length [57]. This third strand remains bound to the L-strand after synthesis and forms a stable d-loop structure [57,58]. This DNA is often called the called the 7S DNA based on sedimentation properties, though it can vary greatly in length depending on which of three 5′ origin sites it originates from [59,60]. The d-loop spans only a portion of the non-coding region, and only some mtDNA molecules contain the structure [61]. The proportion of molecules having the d-loop structure varies by species and cell type, ranging from as low as 1% in rabbit skeletal muscle to 95% in frog oocytes [62,63].

The d-loop sequence contains at least one conserved region and 2 hypervariable regions that offer potential for barcoding. Conserved sequence block 1 (CSB1) is present in most species studied to date, making it a useful “universal target.” While present in humans, CSB2 and CSB3 are often either partially present or missing depending on the species, making their use more application dependent [28,29]. The noncoding region is home to three of the most mutable regions in the mtDNA, and two of these (HSV1, HSV2) overlap with the d-loop [57]. CSB1 offers a tantalizing target for near-universal barcoding, while the hypervariable regions offer enough interspecific diversity due to higher mutation rates that they are capable of separating recently diverged species [30]. Alternatively, species-specific primers targeting the hypervariable regions may offer a quick way to confirm specimen identity or target individual species of interest.

## 3. Diagnostic Markers: Nuclear Genes and Repetitive DNA Elements

### 3.1. Nuclear Ribosomal DNA

While utilized less frequently than mtDNA for vertebrates, rDNA can be a powerful tool for identifying bloodmeal source. rDNA is present in the genome of all eukaryotes in a series of tandem repeats consisting of two rapidly evolving internal transcribed spacers (ITS) sandwiched between extremely conserved coding sequences for 18S, 5.8S, and 28S genes [64,65]. The ITS regions are so divergent in both sequence and length that they can be used to separate lineages within species, but challenging for alignment of sequences from divergent taxa [66,67,68]. The locations and copy numbers of rDNA tandem clusters also vary greatly among eukaryotes [69,70]. However, despite the differences in chromosomal location, the genetic organization of the rDNA loci themselves is remarkably conserved in the vast majority of eukaryotes [64]. Nuclear rDNA markers may be of particular use for species that do not commonly feed on vertebrates. *Uranotaenia sapphirina*, for example, makes a habit of feeding from annelids [71], which may cause assays utilizing other markers to give negative or ambiguous results. Bloodmeal identification may ultimately be realized in these cases using broad-spectrum nuclear targets.

### 3.2. Prepronociceptin (PNOC)

The first human, mouse, and rat sequences for PNOC were published in 1996, revealing conservation among species [72]. The nuclear PNOC gene encodes nociceptin and other neuroactive peptides, and transcription of the gene is predominantly found in the brain and spinal cord [72]. However, there is a region of lower homology in the core of the gene that contains an insertion with a variable number of repeated amino acid motifs that appear to be species-specific that could be useful in the absence of resolution by other markers [72].

Several limitations associated with using PNOC as a barcoding target have been noted, perhaps resulting in its limited usage in the field. Direct comparison between Cyt b and PNOC for bloodmeal identification saw greatly reduced sensitivity of PNOC, a lack of comparative sequences in GenBank, and inadequate resolution of samples at the species level [73,74,75]. While there still may be some use for PNOC confirmation of species that cannot be resolved using other markers, it is unlikely to replace the mtDNA genes as the barcoding target of choice on a broad scale.

### 3.3. Alu Transposable Elements

For studies specifically targeting primate bloodmeal sources, the use of Alu elements can be useful. This large family of transposable elements with copy numbers in the millions makes a plentiful target for amplification. Evolutionary divergence has resulted in several human-specific Alu elements that can potentially be utilized to differentiate human DNA from that of other non-human primates. However, these elements have a lower copy number and so sensitivity may be reduced [76]. Originally used for forensic applications, humans have been identified as the host of phlebotomine sand flies using Alu Yb8 elements [77,78,79].

### 3.4. Other Nuclear Elements

While nuclear gene regions for targeted sequencing are popular in the fields of plant and fungal barcoding [80,81,82], they have not gained traction in vertebrate barcoding, with very few exceptions [83]. Microsatellites, which are generated by polymerase slippage during replication, have remained in use for intraspecific delimitation. Microsatellite DNA can differentiate different family groups within a species as the odds of two unrelated individuals having the same pattern of slippage is very low [84,85]. Microsatellites are typically utilized for studies of multiple-host feeders where identification of individual animals, such as those within a population of endangered species, is critical for understanding the dynamics of host preference and vector–host interactions [85,86,87,88].

## 4. Molecular Advances

A number of advances have been made over the last decade in both technologies and techniques that have introduced new platforms and opportunities for molecular identification of arthropod bloodmeals. While it is easy to dismiss older strategies as obsolete as we embrace “flashy” advancements, the cost effectivity and accessibility of “tried and true” assays have kept these in use by many laboratories across the globe. Fairly novel a decade ago, nucleotide-based approaches have cemented their place as the modern “gold standard” of arthropod bloodmeal identification techniques and the introduction of a plethora of high-fidelity enzymes and improved reagents at a fraction of the cost has made these techniques more accessible. Published PCR assays for bloodmeal identification target a multitude of mitochondrial and genomic loci, though the taxonomic coverage for any one amplicon can be extremely variable. Novel PCR assays have been published for COI [89,90,91], Cyt b [92,93,94,95], 12S and 16S [14,16,96,97,98,99], the d-loop [15], and PNOC [74,75,100,101,102,103], adding to the options available previously (Table S1). In addition to PCR, recent studies have been published that use ELISA [104,105,106,107], restriction fragment length polymorphism (RFLP) [108,109,110,111,112,113], chemiluminescence [114], and agarose gel diffusion [115]. However, since significant advancements have not been made for these technologies and they were thoroughly reviewed by Kent 2009, no additional discussion at this time is necessary; henceforth, we describe, and discuss novel advancements (Table 1).

### 4.1. Real-Time PCR and Quantitative PCR (qPCR)

Still in its infancy in 2009, many advances have been made to real-time PCR technologies in the last decade that have led to their adoption as the “gold standard” of nucleic acid quantification [116]. Real-time PCR offers a high throughput solution that is sensitive, specific, rapid, accessible, and less expensive than in the past. Real-time PCR measures the fluorescent output of fluorophore molecules using an extremely sensitive fluorimeter following sequential rounds of PCR amplification [147]. While the terms “real-time” and “quantitative” are commonly used interchangeably, it is important to distinguish the two assays since they have different data output. Real-time assay results determine whether a sample is positive or negative based on the Ct value in relation to the calculated C_q_. In order for an assay to be called quantitative PCR (qPCR), a standard curve must be generated by concurrently running a series of “standards,” or controls, which subsequently tie the samples of unknown value to a calibrator with a known value. From this curve, the samples can be quantified and relative copy number determined. Real-time and qPCR technologies, suggested terminology, and important information for publication of real-time data have been thoroughly discussed by Bustin et al. [147].

More sensitive than standard PCR, real-time PCR is perfectly suited for the identification of miniscule amounts of host DNA present in arthropods [117,118]. Ideal amplicon size ranges between 75 and 200 nucleotides in length, and this small size can accommodate degraded DNA found in digested bloodmeals. Correct determination of C_q_ greatly reduces rates of false positivity and detection of contaminating DNA introduced during laboratory processing, making qPCR more accurate and specific than nested PCR [148]. However, quantification of samples assumes that the amplification efficiency of the samples and standards are the same, and differences in efficiency can lead to inaccuracies as high as 42.5% [116]. This is of particular concern in multiplexed assays in which the standards may be mixed or mismatched to the sample DNA, yielding inaccurate results. Development of specific sets of standards for each anticipated sample can be impossible when dealing with the unknowns of field-caught arthropod bloodmeal source. Depending on the reagents chosen, primer and probe design, and the number of samples to analyze, the costs may still remain high per sample.

Most real-time PCR assays harness multiplexed primer sets designed specifically for each species, genus, or family of animals anticipated to be the host of the arthropod in question [119]. These assays are limited by primer sets selected in advance to amplify the DNA of known hosts; they are not useful for detection of blood from uncommon or unexpected hosts. Regardless of this limitation, recent studies utilizing real-time PCR technology were able to easily identify multi-host bloodmeals from mosquitoes and sand flies [94,149,150], and assays proved to be extremely specific and had limits of detection below one picogram of DNA for flea bloodmeals [151]. Interestingly, the bloodmeal hosts of ticks were detectable for up to 8 months after feeding, though in a low percentage of samples [14]. A high-throughput survey of *Culicoides* midges identified the meals of 95% of midges examined much more efficiently and cost effectively than PCR and sequencing [152]. This technology has also helped researchers broaden the range of anopheline mosquitoes thought to be capable of malaria transmission to humans [153] and has increased our knowledge of biting behavior and dispersal of mosquitoes in the field [154].

### 4.2. High Resolution Melting Analysis (HRM)

The combination of real-time PCR with melt curve analysis was first introduced in 1997 [155], and higher resolution melting curves were introduced just six years later due to improvements in instrument precision and sampling frequency [120,121]. HRM now offers an incredibly sensitive, simple, and fairly inexpensive solution for bloodmeal analysis. At its core, HRM is a simple real-time PCR assay with an added melt curve at the end of the run using a specific saturating dye [121]. The saturating dye intercalates the double-stranded DNA amplicons and as the temperature is incrementally increased, the denaturing amplicons release the dye which is read by the instrument’s fluorimeter at specified intervals. The data are compiled to produce a disassociation or melt curve which is highly specific to DNA molecule sequence, length, and GC content [121,122].

In addition to simplicity, the biggest benefit of HRM is that it requires no additional instrumentation in addition to a real-time PCR thermal cycler since most modern instruments include the ability to program HRM [122]. A single assay with universal primers can be used to identify bloodmeal source, including mixed sources, since each unique DNA amplicon will generate a unique melting curve [99]. HRM analyses are often more sensitive for smaller fragments in the 150 to 250 nucleotide range, which is useful for degraded DNA [121]. In addition, the technique is not destructive to the denatured amplicons; the amplicons will reanneal upon reduction in the temperature and sequencing can then be performed on the PCR product [122].

The biggest downside to the use of HRM analysis is that the generated curves are highly specific to the sample and qPCR chemistry, so there is currently no standard database of melt curves for comparison. Laboratories must either generate a library of melt curves for comparison in advance by anticipating potential meal source or submit amplification products for confirmatory sequencing after the curves are generated to identify the animal the curve represents. Preparation of a comprehensive library in advance can be difficult for arthropods with a wide range of bloodmeal sources, and sequencing adds time and cost to the project. In some cases, the generated curves may overlap even for unrelated species due to similarities in DNA composition, methylation, or other variables, reducing the sensitivity of the assay [123].

Originally developed to rapidly identify variants in the human genome, this technology is now applicable to a wide range of other uses including the identification of arthropod bloodmeal source [120]. Published assays to date have used either a single gene [156] or a multigene approach [99,123] for the comparison of species-specific melting curves. The assays were sensitive enough to amplify DNA in the meals of triatomine kissing bugs a full thirty days after feeding, specific enough to identify mixed bloodmeals, and capable of identifying meals from field-caught arthropods [99,123,156]. While some overlap of HRM profiles from disparate species was found using a single gene approach, targeting multiple genes for comparison allowed for adequate separation of species [99,123]. Assays have been developed to rapidly identify field-caught fleas that had fed on potentially infectious rodents or shrews and humans up to 72 h after feeding [157,158], helping to estimate plague risk in Uganda. Similarly, studies of triatomine kissing bugs identified risk factors for transmission of *Trypanosoma cruzi*, the causative agent of Chagas disease, and was capable of rapidly estimating insect parasitism within local human populations [159,160]. However, in a bid for further efficiency, it was found that use of automated DNA extraction equipment (KingFisher, ThermoFisher Scientific, Waltham, MA), decreased identification success of bloodmeals from field-caught tick nymphs from around 55% of samples to just 22% [161].

### 4.3. Digital PCR (dPCR) and Droplet Digital PCR (ddPCR)

Digital PCR technology relies on the same principles as standard PCR; however, the mixture is partitioned into micro-reactions before amplification on a thermal cycler. These isolated microreactions reduce template competition for enzymes and primers which improves the efficiency of the reactions in many cases [124]. After a series of amplifications, the ratio of positive partitions to negative partitions is determined using a sensitive fluorimeter, and this is used to quantify the product using Poisson’s statistics [162,163]. It is called “digital” because a single quantification happens at the end of the reaction which reduces the error-prone series of analog quantifications during the exponential phase of real-time PCR into binary (positive or negative) digital signals for each partition [124]. dPCR also eliminates the need for a standard curve, avoiding the uncertainly associated with variations in reaction efficiency between samples and standards [116].

Originally designed to be used with microtubes [164] or 384-well plates [165], advancements in device miniaturization and microfluidics have gone hand in hand with dPCR development. Progress in the field of microfluidics has increased the efficiency of isolation by using massively parallel sample partitioning [166], and advances in microelectronics have increased the number and quality of partitions available in manufactured devices [166,167,168]. An alternative to microplate assays, droplet digital PCR (ddPCR) uses droplets emulsified in specialized oil instead of physical partitioning [169,170,171,172]. Each of the thousands of generated droplets undergo cycles of PCR and are then carefully interrogated sequentially using a process similar to flow cytometry. Droplets suspended in oil are very delicate and easily subjected to shearing forces, potentially leading to cross contamination or destruction of a portion of the droplets before reading. To address this issue, partitioning can be performed using magnetic beads that attract a single DNA molecule to each individual bead but allow for coating with the amplified product. These cytometry-friendly beads are more stable and are not subjected to the same damage due to shearing forces, so the beads may be identified by flow cytometry at very high throughput [124]. dPCR technologies and platforms were thoroughly reviewed by Quan et al. in 2018, including ddPCR [124].

While digital technologies are promising, there are a number of limitations. A number of very specialized pieces of equipment must be added to the laboratory, and many are expensive. Depending on how effective the selected partitioning equipment is, the number of partitions selected, and the desired volume of the partitions, qPCR may still be more sensitive and less expensive per sample than dPCR [124]. The accuracy and sensitivity of dPCR are dependent on the number of partitions and variations in volume in those partitions, making both of these variables extremely important in determining the utility of the assay [124]. Sensitivity is also highly dependent on the rates of false positive and false negative partitions, making solid design, validation, and optimization critical to performance [125]. This can result in a high cost upfront for validation. Currently, droplet readers are only capable of reading two fluorescent colors at a time, meaning multiplexing of reactions is extremely limited. If consensus primers are used, additional PCR and sequencing must be performed to confirm the identity of the sample, leading to additional costs and precluding the use of this technology for precious small volume samples.

While digital technologies have been applied to the identification of arthropod-borne pathogens [173,174,175,176,177,178,179], to date only one published study using ddPCR techniques has been applied to the identification of arthropod bloodmeals [180]. The ddPCR assay was marginally more sensitive than traditional PCR, amplifying bloodmeal DNA up to 48 h after ingestion [180]. This experiment showed the utility of single target ddPCR for sensitive amplification of trace amounts of DNA in an artificially fed arthropod. However, to date there are no studies utilizing this technique for field-caught arthropods.

### 4.4. Next Generation Sequencing (NGS)

Technological advances in NGS have allowed both deep sequencing and shotgun-style metagenomics to be used more widely [127,181], and these advancements have been reviewed thoroughly elsewhere [182,183,184,185,186]. Metabarcoding approaches paired with NGS to determine the species represented in mixed biological samples offers a tantalizing opportunity to mine vast amounts of sequence data from individual samples, including identification of pathogens, hosts, and vector from a single NGS run [187]. Unlike traditional PCR where primers must be designed in advance, NGS can use random primers during preparation of cDNA that allow for sampling of the true diversity of arthropod bloodmeals because the amplification is not biased by primer design [126,127]. Even when specific primers are used, the depth of coverage and accuracy offered by this technology can often differentiate the sequences of nuclear mitochondrial DNA (NUMTs) from mtDNA, clarifying species identification for mtDNA targets [128].

Intelligent design of sample handling, library preparation, and data processing pathways limits waste and helps keep sequencing costs lower. Massively multiplexed runs were made possible by the introduction of indexed adapters with unique barcodes, allowing large numbers of samples to be pooled into libraries for simultaneous sequencing. Targeted enrichment before NGS can also be beneficial for enhancement of the number of useable sequences that come from a run. Enrichment of microscopic quantities of DNA present in bloodmeals can be carried out by PCR amplification of desired loci to increase the number of sequencing templates available for NGS [188], or by use of specially designed probes to enrich arthropod, bloodmeal host, and pathogen DNA [189].

Despite exciting advances in NGS technologies, there are a number of shortcomings to consider. NGS provides a truly immense amount of raw data, making specific training in bioinformatics and command line computing environments a necessity for data analysis [129]. The number of choices that need to be made for library preparation can be daunting to those new to the field, and each specific application can have radically different procedures. Much like other forms of sequencing, challenges still exist with reference sequence availability, taxonomic resolution of the target gene, primer bias, and arthropod bloodmeal degradation. However, perhaps the largest barrier to NGS use is the fact that instrument costs remain inaccessibly high and per sample processing costs remain higher than other techniques, excluding many laboratories from harnessing NGS in their research. Some instrument advances are addressing cost issues and are becoming increasingly competitive, as discussed below in Advances in Portability and Field-Forward Technologies.

To date, few studies have applied NGS technologies to the identification of bloodmeals taken by field-collected arthropods. The assays that have been applied showed that NGS is sensitive enough to amplify minute traces of DNA from mosquito midguts [189] and accurate enough to source multiple-host mosquito and kissing bug meals correctly [127,188]. The sequence data provided is detailed to the point of being capable of differentiating individual human hosts, and one study showed that 5% of anopheline mosquitoes in a study area in Papua New Guinea unambiguously fed on more than one human [127]. These data have big implications for human disease transmission, and this level of detail could not be provided by traditional PCR and Sanger sequencing.

### 4.5. Microarray and Microsphere Assays

To address the need for cost effective and high-throughput methods, microarrays and microsphere-based molecular technologies were developed to identify common and predicted bloodmeal hosts [130,131,132,133]. Microarrays and microsphere assays rely on fluorescent or electrochemical detection and can be formulated for either chip-based solid-phase arrays or microscopic polystyrene beads. A series of unique capture probes are designed to be specific for target sequences of interest, and either a chip is synthesized with probe-coated microspots or beads are coated in individual specific primers [190]. Up to 100 unique probes can be designed and tested in a single reaction, resulting in extremely high throughput [134]. After PCR amplification of the sample, the amplicons bind their complimentary probe sequence, and the result is release of a fluorophore or electrochemical signal [133]. Chip-based signals are read by a specialized instrument that scans each individual spot using a laser. Similar to flow cytometry, each bead is sequentially interrogated with two lasers: one to identify the bead and the other to measure the fluorescence of the hybridized probe and DNA [130]. The quantity of fluorescence is dependent on how many probes bound DNA molecules; the higher the fluorescence, the greater the quantity of target DNA in the sample [190].

The small size of the probes could potentially be useful for capture of degraded DNA, increasing the sensitivity of detection over other methods requiring long and intact strands of DNA. The biggest downside to this technology is that expected bloodmeal sources must be determined in advance, the sequence of the target host must be available, and the probes must target short loci that are completely unique with no overlap between closely-related species. Even if the probes are well designed and current databases are thoroughly interrogated, the potential for unexpected cross-species detection remains due to the incomplete nature of the databases. Equipment must be added to the laboratory to couple the probes to the microspheres and to read the beads, which can add upfront cost for laboratories wishing to use this technology. Despite these challenges and although it has only been applied to mosquito meals to date [130,131,132,133], this technology has potential for application across arthropods.

### 4.6. Mass Spectrometry

In recent years, multiple mass spectrometry techniques have been applied to arthropod blood meals. Liquid chromatography tandem mass spectrometry (LC-MS/MS) has been used with both targeted and non-targeted proteomics approaches to identify microscopic quantities of host proteins present in blood fed arthropods. When paired with shotgun proteomics, LC-MS/MS can identify the source of bloodmeals from the entire detected proteome using a genome-free, non-targeted proteomics platform and spectral matching technologies [135,136,137,138,139,140]. Önder et al. published an extensive protocol for use of this method in 2014 [135]. Alternatively, LC-MS/MS specifically targeting the most abundant protein present in bloodmeals, hemoglobin, was pioneered for use in ticks and triatomine kissing bug meals [141,191,192]. The instrument generates a spectrum for the sample which can then be compared to theoretical spectra generated from known protein sequences to determine the sample sequence [193]. This can be compared to publicly available DNA and protein sequences in databases to determine the identity of the sample [141]. Sequencing of hemoglobin proteins offers a great potential for success where nucleotide-based techniques fail because hemoglobin is an extremely stable molecule; remnants of hemoglobin derived porphyrins were still detectible in a mosquito from the middle Eocene period using this technique [194]. Quantitative LC-MS/MS techniques can quantify absolute amounts of hemoglobin by comparison to a stable isotope standard using AQUA [195], and for those arthropods that commonly take multiple bloodmeals, absolute concentration of the hemoglobin from each of those meals can be determined [195,196].

Although this is an extremely promising technique, especially for degraded bloodmeals, this technique is limited by the robustness of sequence databases much as nucleotide-based techniques are. There is some variability in hemoglobin across vertebrates and some species may have polymorphic hemoglobin peptides while other sequences are conserved across multiple species, leading to some reduction in resolution for some targets. LC-MS/MS instrumentation is a substantial investment and so these techniques may be inaccessible for many facilities lacking a dedicated proteomics core, and sample processing currently can range between USD 10 and USD 100 per sample.

Matrix-assisted laser desorption/ionization time-of-flight mass spectrometry (MALDI-TOF MS) has recently come into use to identify both arthropods and the source of their bloodmeals [144,197,198]. MALDI-TOF MS relies on a matrix of proteins that are ionized and propelled in a flight tube by their mass-to-charge ratio [143], and the resulting output is a spectrum profile highly specific to the sample [199]. Samples can be processed and results retrieved with extreme rapidity, often in under one hour [198], and data analysis is simple with currently available software [142]. The spectra generated by this technology have been found to be specific to species, capable of discerning even between closely-related primates and felines [142,143].

While this promising technique is reported to be less costly than nucleotide-based assays despite requiring expensive instrumentation, it also has the major limitation that no commonly accessible, searchable database of profiles currently exists. Most studies utilizing this technique create “home-made” libraries limited to arthropods engorged on specific hosts of interest [142,144], which limits the usefulness of MALDI-TOF MS application for the study of the feeding habits of field-collected arthropods [200,201]. However, this current limitation is not insurmountable. The Bruker MALDI Biotyper offers a precedent for the ability to identify microorganisms in minutes, and the broad utility this technology could be applied to arthropod blood meals. Similar to PCR, issues with degradation of bloodmeals has been reported and spectral degradation can result in less robust identification [143,144]. The storage method can also greatly influence the quality of the profiles generated, so sample conditions must be chosen with care [144]. It has been noted that profiles generated by blood loaded directly for analysis and blood from mosquito abdomens were not superimposable, indicating some changes to the blood proteins in the arthropod [142,144]. Finally, while the costs of sample preparation and running the samples are very low, instrument costs remain high, making it a costly addition to a lab not actively using MALDI-TOF MS for other applications. Despite these challenges, this technology has been used to identify the bloodmeal sources of ticks [198,202], mosquitoes [142,143,144,199,200,201], and *Culicoides* midges [197].

### 4.7. Stable Isotope Analysis (SIA)

To address the highly problematic and extremely short window in which the undigested contents of a bloodmeal can be molecularly identified, stable isotope analysis has been employed to identify completely digested bloodmeals [145]. Long used in food web analysis, the ratios of stable isotopes such as carbon (^13^C/^12^C) or nitrogen (^15^N/^14^N) are utilized to differentiate animals based on their diet [203,204,205,206]. There are two primary assumptions critical for employment of this technique, the first is that each host species has a unique diet in nature and the second is that the unique mechanisms of each animal’s metabolism can result in different isotopic ratios even if the diet is similar [146]. This methodology relies on gas isotope-ratio mass spectroscopy which involves burning samples to create a gas, ionizing the gas, and separating the ionized sample by magnetic field according to mass [205]. The results are then compared to a library of SIA signatures developed from potential host species. Comparison of several isotopes, including nitrogen, carbon, hydrogen, oxygen and sulfur, for example, may increase resolving power [207].

Proof of concept experiments were able to separate human- or chicken-fed mosquitoes one full week after ingestion of the bloodmeal [145], and gerbil- or rabbit-fed ticks after molting [208]. This methodology was applied to wild caught ticks [146,209], fleas [210], and mosquitoes [211] with mixed results. While this technique is theoretically capable of determining meal source long after digestion of the bloodmeal giving it great potential for use across arthropod species, specificity and species resolving power remain a challenge. Animals with similar diets, such as mice and chipmunks, were incapable of being distinguished using SIA [146]. SIA signatures can vary greatly for the same species among different habitats during different seasons because of the differences in feeding patterns [146,212,213], leaving researchers to create specific, local, and temporal libraries for comparison. Despite these challenges, the ability to potentially identify meal source after long periods of time remains a tantalizing opportunity for bloodmeal identification and, as SIA remains in its infancy, technology advances may address current issues.

## 5. Advances in Portability and Field-Forward Technologies

Perhaps the most universally beneficial advances for field studies of arthropods have come in form of miniaturization and the development of field-forward platforms that can follow the researcher into the harsh conditions of field research. While some commercially available mobile laboratories were available a decade ago, these typically consisted of full-sized laboratory equipment packaged into a heavy case and used with field-adapted protocols [214]. More recently, powerful miniaturized laboratories designed specifically for field deployment have come into vogue. Perhaps the most notable, the MinION next generation sequencer (Oxford Nanopore Technologies, Oxford, UK) weighs in at less than a hundred grams, can fit in your pocket, and currently costs less than USD 5000. Powered by USB, this nanopore sequencing instrument is capable of up to 20 GB of long read sequence data that can provide more information for species differentiation [215]. The MinION has been transported all over the globe from rainforests to the artic [216], was deployed to Brazil to help with Zika virus surveillance during the 2016 outbreak [217], and has even been tested on the International Space Station [218]. To date, use of this platform has been commonly applied to pathogen discovery and identification [219], but has also demonstrated efficacy in Cyt b sequencing from wildlife samples [220] which may also imply utility for field-based arthropod blood meal identifications. Furthermore, the MinION holds great potential for simultaneous identification of arthropod, pathogens, and bloodmeal source. This technology eliminates the complications associated with sample storage, including freeze/thaw cycles that can damage precious pathogen and host DNA in arthropod bloodmeals, making it a valuable addition to the technological arsenal available for research studies [216].

Another recent and useful addition to the array of field-forward equipment is the Franklin mobile qPCR thermocycler (Biomeme Inc., Philadelphia, PA, USA). This instrument can run for a full day on a single battery charge, sends and syncs results to a cellular phone or tablet via an onboard Wi-Fi or cellular data transmitter, weighs in at just two pounds, and can deliver the results of a presence/absence assay in one hour [221]. In addition to the equipment, a pre-packaged sample preparation kit uses syringe mounted extraction columns and color-coded reagents to prepare the sample, and pre-packaged lyophilized, shelf stable qPCR “Go-Strips” can be used to run the assay, precluding the necessity for heat blocks, centrifuges, or cold storage [222]. Originally designed to be a point-of-care diagnostic tool, the instrument has been field tested alongside the MinION in the detection of arboviruses in pools of field caught mosquitoes [219]. Older Biomeme units have been deployed for use in foot-and-mouth disease detection in the field [223], as well as point-of-care detection of canine distemper virus [222]. The very same capabilities can easily be put to work for bloodmeal identification.

Similar to the Franklin but new to the scene in early 2020 is the bCUBE miniature portable DNA testing laboratory (Hyris Global Diagnostics, London, UK). This Wi-Fi and ethernet enabled app-based thermal cycler was designed for ultimate portability, weighing in at 1.15 kg and measuring only 10 × 10 × 12 cm. The bCUBE can run qRT-PCR analyses using FAM and HEX fluorophores alone or in tandem and can conduct high resolution melting analysis concurrently. Paired with a cloud-based app, this device can be used anywhere on the globe for rapid field identification of RNA and DNA samples. Currently, the bCUBE offers bKITs for analysis of sixteen plant, bacteria, or pathogen samples at a time, though the company is open to expanding their offerings. The bCUBE has been tested for laboratory identification of dengue and Zika viruses in infected mosquitoes [224] and point of care diagnosis of the novel coronavirus (SARS-CoV-2) [225]. While it has not yet been applied to arthropod bloodmeal identification, there is great potential for identification of arthropod blood meals at the site of field collection, though the limitation on number of samples will not make this a high-throughput option.

PCR instrumentation is not the only technology to have found recent field-forward advances. The World Health Organization (WHO) has put an emphasis on development of point-of-care tests that are affordable, sensitive, specific, user-friendly, robust, rapid, equipment-free, and deliverable (ASSURED) [226]. This has sped development of technologies for disease diagnosis that have potential use for arthropods in the field. Microarray platforms have been developed to address the needs for rugged and miniaturized field deployable equipment in sizes small enough to allow for easy transportation [133]. The ElectraSense Reader (Custom Array, Bothell, WA, USA) was used in the field in temperatures up to 35.6 °C and humidity to 92% in Southeast Asia and was able to identify common host sources of mosquitoes [133]. Additionally, lab-on-a-chip (LOC) devices allow for sample preparation, analysis, and results on a single, enclosed device, reducing the number of reagents that must be hauled into the field [227]. LOC also allows for smaller sample volume necessary, reduces the potential for contamination, allows multiplexing, and increases the throughput of samples [227]. While LOCs have been deployed for rapid blood analysis [228] and genotyping parasites such as malaria [229], it has not to date been applied to arthropod bloodmeal analysis, but it is only a matter of time before assays are developed and deployed to the field.

## 6. Xenosurveillance and Other Applications

From a public and veterinary health perspective, understanding the disease burden on the human or animal population at a given place and time is an important component of designing targeted surveillance and mitigation strategies. Obtaining blood samples from humans or other animals for diagnostic purposes is not always easy. Confronted with this challenge, Grubaugh et al. [230] employed the innovative approach of using engorged mosquitoes to survey the pathogens circulating in the community. This approach of “xenosurveillance” refers to the technique of exploiting the hematophagous behavior of some arthropods to survey vertebrates for the presence of infectious disease agents. Capitalizing on the anthropophilic feeding patterns of *Anopheles gambiae* s.l. mosquitoes, teams aspirated engorged mosquitoes from huts in West Africa and dried blood spots on FTA cards for downstream processing [230]. Analysis of the nucleic acids extracted from these cards revealed not only the vertebrate host of the mosquito, but also pathogens circulating in that individual. Laboratory optimization studies demonstrated the proof-of-concept that viral, bacterial, and parasitic agents can be detected from the few microliters of blood that comprise a mosquito bloodmeal [230,231]. Field validation of this technique has so far identified the DNA of Epstein Barr virus in a mosquito bloodmeal from a person, and canine distemper virus in a mosquito bloodmeal from a dog [230]. The analogous “iDNA” method has similarly employed the analysis of DNA from engorged leeches and hematophagous arthropods to assess wildlife diversity in addition to host-vector associations [232]. Antibodies against pathogens circulating in a vertebrate population can also be assayed using a xenosurveillance approach [233], further increasing the information that can be obtained on the ecology and circulation of a disease agent from surveillance of the vector population.

In addition to xenosurveillance, there are many other questions that bloodmeal analysis has the potential to answer. Meals taken from zoo animals have been used to assess the flight distance of mosquitoes trapped after feeding [234,235], making use of the unique nature of the meals to calculate the distance traveled. Bloodmeals can be utilized to assess the biting rates of arthropods, allowing for better calculation of disease risk in communities [236]. Assessment of calculated protection rates can be determined by investigating the numbers of mosquitoes that have fed on humans in homes using bed nets for the prevention of arthropod-borne diseases [237]. Determination of the number of bites taken per gonotrophic cycle can drastically change the transmission risk of malaria parasites by *Anopheles gambiae* vectors, leading to updated and more accurate calculations [238]. The potential applications of bloodmeal analysis are endless, bound only by the accuracy of technologies employed and experimental design.

## 7. Lessons Learned over the Past Decade

While the benefits of using mtDNA for barcoding life and identifying arthropod bloodmeal sources are numerous and myriad studies have successfully used available assays targeting mtDNA, Hebert et al. [33] issued a warning that should be recalled: “It is, of course, impossible for any mitochondrially-based system to resolve fully the complexity of life.” Previously, it was broadly assumed that mtDNA evolves between three and five times faster than nuclear DNA (nuDNA) resulting in higher taxonomic resolution [239]. More recent studies have determined that the ratio of mtDNA to nuDNA mutation rates varies significantly among taxonomic groups [240]. Scaled reptiles and birds have mtDNA mutation rates 26.4 and 24 times higher than nuDNA, respectively, and high rates of mtDNA mutation could potentially lead to greater intraspecific variation, reducing or closing the “barcoding gap” between interspecific and intraspecific variation [240,241]. Many studies utilize the generic guidance that sequence identities ranging from 98–100% identity qualify as conspecific, those ranging between 91–97% are congeneric, and those ranging between 88–90% identity are confamilial, and this remains the best estimate at present [2,33,242]. Caution must be applied to barcoding the source of bloodmeals since the intraspecific variation of many targets of interest may fall outside of these values. Meier et al. [243] argues that common calculation methods for determining barcoding gaps that use the mean interspecific variation instead of the smallest interspecific variation value are overestimating the barcoding gap and this makes speciation more difficult. Ultimately, the determination of species rests with the diligence of the study author, who would do well to educate themselves on the pitfalls determining cut-off values and adjust according to their target organisms.

Unambiguously identifying the source of vector bloodmeals to the species level is necessary for the evaluation of host-vector interactions in a number of vector-borne disease systems. Early critics were quick to point out that potential limitations of using mtDNA to infer species boundaries include incomplete lineage sorting, sex-biased gene flow, selection on mtDNA since the whole genome is one linkage group, introgression, paralogy resulting from transfer of mtDNA gene copies to the nucleus (NUMTs and pseudogenes), ancestral polymorphisms that persist long after species divergence (incomplete lineage sorting), and historical introgression of mitochondria between species potentially dating back millions of years [239,244,245,246,247]. Recent studies have shown these to be of concern in multiple phyla of potential interest in the sourcing of arthropod bloodmeals, including deer of the genus *Odocoileus*, elephants, dolphins, bovines, and freshwater fish [248,249,250,251]. In some of these cases where speciation is the goal, other mtDNA, nuDNA or ribosomal RNA targets may need to be utilized for definitive results. Studies may begin general speciation of bloodmeals using a common target such as COI or Cyt b, but then may add additional markers for further clarification of the target organism as necessary.

As a result of more widespread barcoding, the number of sequences housed in the GenBank database [252] and BOLD [37] has grown exponentially and coverage across all kingdoms has greatly improved. While this is generally a great benefit to researchers identifying a large range of arthropod bloodmeal hosts, this increase in submissions has allowed deposition of some small yet unknown percentage of erroneous sequences [253]. Curation of open databases relies on accurate data submission by the scientist, and some phenotypically difficult-to-identify specimens may accidentally be submitted by researchers lacking the taxonomic expertise to perform the identification [254]. Incorrect sequences or metadata, including mislabeled sequences belonging to contaminating bacteria, have been submitted accidentally [255], and missing sequence information for rare, hard to sample, or endangered specimens can hamper identification efforts [256]. As the databases grow, aging sequence data containing outdated taxonomy associated with outdated submissions remains.

Despite these potential pitfalls, very few surveys have been conducted to determine the burden of erroneous sequences. A study of metazoan sequences in GenBank found the maximum estimate of the error rate increased with decreasing taxonomic rank [257]. At the genus level, 55% of errors identified were the result of a change in taxonomic status from the time of entry until the time of search [257]. Some researchers have undertaken efforts to resolve errors by scanning the databases to identify erroneous sequences, which can be done by identifying instances in which intraspecific Kimura 2-parameter (K2P) variation exceeded interspecific K2P divergence, or by identifying high levels of intraspecific K2P variance [33,254,258]. Studies of fish and turtle sequences were hampered by shallow interspecific divergence and high intraspecific divergence, as well as outdated taxonomies associated with the sequences [33,253,254,258]. Because of these complications, no concrete system has yet been devised to root out and update errors within the databases. The use of curated databases, such as BOLD, and recent improvements and attention to quality control of the GenBank database will reduce these error rates over time but results that appear out of expectation for bloodmeal source should be closely scrutinized.

## 8. Conclusions

Due to the scientific and technological advances of the last decade, the options available for molecular identification of arthropod bloodmeals have greatly increased. A better understanding of the factors effecting the utility of certain loci has allowed researchers to harness more powerful data collection useful to research objectives. Technology advancements have focused on developing sensitive, specific, rapid, accessible, and high-throughput solutions that are capable of differentiating even closely-related species. Some of these advancements have come in improving existing technologies, such as next generation sequencing, real-time PCR, and digital PCR, or making these more accessible to laboratories through decreasing costs. New technologies have opened new doors to alternative identification methods, including use of non-nucleotide-based techniques like mass spectrometry and stable isotope analysis, many of which are promising. Despite these advances, many of the same factors affecting the decision-making process come into play: cost, accessibility, equipment, manpower, and time.

The largest and most ubiquitous challenge to identification of arthropod bloodmeals remains coaxing information out of digested and heavily degraded bloodmeal materials. This challenge is twofold: degradation prior to sampling and degradation after the sample has been obtained. For most nucleotide-based assays, mosquito bloodmeals must be sampled within 36 to 48 h after feeding, leaving a very small window for which the bloodmeal may be sourced [180]. In addition to battling degradation, storage of the materials presents a challenge to avoid further issues with DNA integrity and storage conditions must be carefully chosen to suit the needs of the research [259]. Many of the advances discussed here such as ddPCR and mass spectrometry technologies, as well as assays targeting equally informative but smaller and more stable DNA regions, have specifically tried to address the needs of accurate identification of degraded materials. While some such as ddPCR have yet to offer improvement, those targeting longer-lived molecules such as hemoglobin do hold promise for improvements should the assays be further developed and optimized.

Advances in miniaturization have allowed the power of the laboratory to be delivered directly to the field, such as through the MinION, Biomeme, and bCUBE portable devices. These technologies meet the World Health Organization’s push for affordable, sensitive, specific, user-friendly, robust, rapid, equipment free, and deliverable (ASSURED) point-of-care tests and may change the way that field samples are processed in the future. These platforms also allow for processing of samples without the need for cold storage or stabilization, potentially addressing some sources of degradation.

Continued advancements and research in the fields of vector–host interactions, host preference, and disease ecology present opportunities for valuable data collection and filling existing gaps in knowledge. This research will give us a better understanding of hematophagous arthropods and their unique behaviors, allowing us to build more accurate predictive models of disease transmission and arthropod ecology.

## Figures and Tables

**Table 1:** The benefits and pitfalls of new technologies being applied to bloodmeal analysis [116,117,118,119,120,121,122,123,124,125,126,127,128,129,130,131,132,133,134,135,136,137,138,139,140,141,142,143,144,145,146].

Technology:	Pros:	Cons:
Real-time and qPCR	- Gold standard of nucleic acid quantification - Sensitive, specific, rapid - Increasingly affordable	- Requires expensive instrumentation and reagents - Requires specific primer sets for anticipated animals
High Resolution Melting	- Compatible with real-time PCR equipment - Specific to DNA sequence, length, and GC content - Unique melt curves generated with universal primers - Useful for small amplicons and degraded DNA	- Requires expensive instrumentation - Must generate a unique library for comparison - Curves may overlap for unrelated species
Droplet Digital PCR	- Sensitive for low copy and rare DNA targets	- Requires specialized equipment - Accuracy and sensitivity are dependent on reaction conditions - Substantial and costly optimization
Next Generation Sequencing	- Target sequence agnostic - Can differentiate between mtDNA and NUMTs - Multiplexing capability - New, field-forward inexpensive portable instruments	- High equipment and sample processing costs - Data analysis requires specialized training - Partially digested DNA cannot be amplified/sequenced
Microarray and Microsphere	- Sensitive and high-throughput - Small probes can capture degraded DNA	- Requires specialized instrumentation - Unique probes must be designed for each target
Mass Spectrometry	- Targets proteins that are more stable during digestion than DNA - Can generate reference library of spectra from known protein sequences	- High equipment and sample processing costs - Relies on a comprehensive reference library - Project-specific reference libraries must be generated - Sample storage can influence profile quality
Stable Isotope Analysis	- Useful for samples with degraded DNA	- High equipment and sample processing costs - Specificity and species resolution remain a challenge - Project-specific reference libraries must be generated

## Supplementary Materials

The following is available online at https://www.mdpi.com/2075-4450/12/1/37/s1, Table S1: Primer sets commonly used to explore bloodmeal source by molecular methods.
