# Supplementary material for: Modernizing the Toolkit for Arthropod Bloodmeal Identification
Source: Insects. 2021 Jan 6;12(1):37. doi: 10.3390/insects12010037 (PMC7825046; doi:10.3390/insects12010037)
Supplement: Supplementary file 1 [file insects-12-00037-s001.pdf]

insects

MDPI

Table S1: Primer sets commonly used to explore bloodmeal source by molecular methods.

DNA Target	Target Locus	Primer Source	Species Design	Size (nt)	Primer Name	Sequence	Assay Use
mtDNA	COI	Ivanova et al. 2007 [1]	Fish, but used for nearly all vertebrates	658	VF1_t1	TGT AAA ACG ACG GCC AGT TCT CAA CCA ACC ACA AAG ACA TTG G	PCR
					VF1d_t1	TGT AAA ACG ACG GCC AGT TCT CAA CCA ACC ACA ARG AYA TYG G	
					VFLi_t1	TGT AAA ACG ACG GCC AGT TCT CAA CCA ACC AIA AIG AIA TIG G	
					VR1d_t1	CAG GAA ACA GCT ATG ACT AGA CTT CTG GGT GGC CRA ARA AYC A	
					VR1_t1	CAG GAA ACA GCT ATG ACT AGA CTT CTG GGT GGC CAA AGA ATC A	
					VRli_t1	CAG GAA ACA GCT ATG ACT AGA CTT CTG GGT GIC CIA AIA AIC A	
mtDNA	COI	Townzen et al. 2008 [2]	Amphibians, mammals, avians	663	Long 5934 FWD	AAC CAC AAA GAC ATT GGC AC	PCR
					Long 6597 REV	AAG AAT CAG AAT ARG TGT TG	
mtDNA	COI	Cicero and Johnson 2001 [3]	Avians	500	L7327	CCT GCA GGA GGA GGA GAY CC	PCR
					H7827	CCA GAG ATT AGA GGG AAT CAG TG	
mtDNA	COI	Reeves et al. 2018 [4]	Terrestrial vertebrates including reptiles	244, 395, 664	Mod_RepCOI_F	TNT TYT CMA CYA ACC ACA AAG A	PCR
					Mod_RepCOI_R	TTC DGG RTG NCC RAA RAA TCA	
					VertCOI_7194_F	CGM ATR AAY AAY ATR AGC TTC TGA Y	
					VertCOI_7216_R	CAR AAG CTY ATG TTR TTY ATD CG	
mtDNA	COI	Nagy et al. 2012 [5]	Reptiles	664	RepCOI-F	TNT TMT CAA CAN ACC ACA AAG A	PCR
					RepCOI-R	ACT TCT GGR TGK CCA AAR AAT CA	

DNA Target	Target Locus	Primer Source	Species Design	Size (nt)	Primer Name	Sequence	Assay Use
mtDNA	COI	Alcaide et al. 2009 [6]	Vertebrates including mammals, avians, reptiles, and amphibians	758	M13BCV-FW	TGT AAA ACG ACG GCC AGT- HAA YCA YAA RGA YAT TGG NAC	PCR
					BCV-RV1	GCY CAN AYY ATN CYY RTR	
				758	BCV-RV2	GCY CAN AYY ATN CYY RTR TAN CC	
					M13-F	TGT AAA ACG ACG GCC AGT	
mtDNA	COI	Parodi et al. 2002 [7]	Humans	228	Human CoxI Fwd	TTC GGC GCA TGA GCT GGA GTC C	PCR
					Human CoxI Rev	TAT GCG GGG AAA CGC CAT ATC G	
			Rodents	150	Mouse CoxI Fwd	ATT ACA GCC GTA CTG CTC CTA T	
					Mouse CoxI Rev	CCC AAA GAA TCA GAA CAG ATG C	
				196	Rat CoxI Fwd	CGG CCA CCC AGA AGT GTA CAT C	
					Rat CoxI Rev	GGC TCG GGT GTC TAC ATC TAG G	
			Monkeys	222	Monkey CoxI Fwd	CCT CTT TCC TGC TGC TAA TG	
					Monkey CoxI Rev	TTT GAT ACT GGG ATA TGG CG	
			Dogs	153	Dog CoxI Fwd	GAA CTA GGT CAG CCC GGT ACT T	
					Dog CoxI Rev	CGG AGC ACC AAT TAT TAA CGG C	
			Cats	180	Cat CoxI Fwd	TTC TCA GGA TAT ACC CTT GAC A	
					Cat CoxI Rev	GAA AGA GCC CAT TGA GGA AAT C	
			Rabbits	151	Rabbit CoxI Fwd	CGG GAA CTG GCT TGT CCC CCT G	
					Rabbit CoxI Rev	AAC AGT TCA GCC AGT CCC CGC C	
			Horses	235	Horse CoxI Fwd	CCC TAA GCC TCC TAA TCC GT	
					Horse CoxI Rev	AGG AAT GAT GGG GGA AGT AA	

DNA Target	Target Locus	Primer Source	Species Design	Size (nt)	Primer Name	Sequence	Assay Use
mtDNA	Cyt b	Cicero and Johnson 2001 [3]	Avians	772	L15557	GAC TGT GAC AAA ATC CCN TTC CA	PCR
					H16065	GGT CTT CAT CTY HGG YTT ACA AGA C	
				1143	L14851	CCT ACT TAG GAT CAT TCG CCC T	
					L14987	CCA TCC AAC ATC TCW GCW TGA TG	
					L15236	TAC CTA AAC AAA GAA ACB TGR AA	
					H15304	GTA GCA CCT CAG AAB GAT ATT TG	
					L15661	ACC TCC TAG GAG AYC CAG AHA AYC	
					H15706	TAT GCG AAT AGG AAR TAY CAY TC	
					H15906	ATG AAG GGA TGT TCT ACT GGT TG	
mtDNA	Cyt b	Boakye et al. 1999 [8]	Vertebrates	358	BM1	CCC CTC AGA ATG ATA TTT GTC CTC A	PCR
					BM2	CCA TCC AAC ATC TCA GCA TGA TGA AA	
mtDNA	Cyt b	Meece et al. 2005 [9]	Vertebrates	358	BM1	CCC CTC AGA ATG ATA TTT GTC CTC A	PCR & RFLP
					BM2	CCA TCC AAC ATC TCA GCA TGA TGA AA	
mtDNA	Cyt b	Townzen et al. 2008 [2]	Amphibians, mammals, avians	457	15150 FWD	GAG GMC AAA TAT CAT TCT GAG G	PCR
					15607 REV	TAG GGC VAG GAC TCC TCC TAG T	
mtDNA	Cyt b	Kocher et al. 1989 [10]	Vertebrates	307	L14841	AAA AAG CTT CCA TCC AAC ATC TCA GGA TGA TGA AA	PCR
					H15149	AAA CTG CAG CCC CTC AGA ATG ATA TTT GTC CTC A	
mtDNA	Cyt b	Latch et al. 2009 [11]	Deer	1246	Odh-cytbF-14153	TCA ATG ACC AAC ATC CGA AA	PCR
					Odh-cytbR-15399	GGG TGT TGA TAG TGG GGC TA	

DNA Target	Target Locus	Primer Source	Species Design	Size (nt)	Primer Name	Sequence	Assay Use
mtDNA	Cyt b	Abbasi et al. 2009 [12]	Vertebrates	344	Cyto1	CCA TCA AAC ATC TCA GCA TGA TGA AA	PCR
					Cyto 2	CCC CTC AGA ATG ATA TTT GTC CTC	
mtDNA	Cyt b	Mota et al. 2007 [13]	Vertebrates	420, 315	DC-cytb-UP	CRT GAG GMC AAA TAT CHT TYT	PCR
					DC-cytb-DW	ART ATC ATT CWG GTT TAA TRT	
					H-cytb-UP	AGG AGA GAA GGA AAG AAG T	
mtDNA	Cyt b	Ngo et al. 2003 [14]	Mammals, avians	772	FWD	CGA AGC TTG ATA TGA AAA ACC ATC GTT	PCR
					REV	TGT AGT TRT CWG GGT CHC CTA	
mtDNA	Cyt b	Kent and Norris 2005 [15]	Specific vertebrates	623	UNREV1025	GGT TGT CCT CCA ATT CAT GTT A	PCR
					UNFOR403	TGA GGA CAA ATA TCA TTC TGA GG	
				453	Pig573F	CCT CGC AGC CGT ACA TCT C	
				334	Human741F	GGC TTA CTT CTC TTC ATT CTC TCC T	
				132	Goat894F	CCT AAT CTT AGT ACT TGT ACC CTT CCT C	
				860	Dog368F	GGA ATT GTA CTA TTA TTC GCA ACC AT	
				561	Cow121F	CAT CGG CAC AAA TTT AGT CG	
					Cow371R	GAG CTA GAA TTA GTA AGA GGG CC	
mtDNA	Cyt b	Fornadel and Norris 2008 [16]	Mammals	98	UNIFORA	TCC AAA CAA CRM AGC ATA ATA TT	PCR
					UNREV1025 [15]	GGT TGT CCT CCA ATT CAT GTT A	

DNA Target	Target Locus	Primer Source	Species Design	Size (nt)	Primer Name	Sequence	Assay Use
mtDNA	Cyt b	Molaei et al. 2006 [17]	Avians, mammals	508	Avian a FWD [3]	GAC TGT GAC AAA ATC CCN TTC CA	PCR
					Avian a REV	GGT CTT CAT CTY HGG YTT ACA AGA C	
				515	Avian b FWD [18]	CCC TCA GAA TGA TAT TTG TCC TCA	
					Avian b REV	CCT CAG AAK GAT ATY TGN CCT CAK GG	
				772	Mammalian a FWD	CGA AGC TTG ATA TGA AAA ACC ATC GTT G	
					Mammalian a REV	TGT AGT TRT CWG GGT CHC CTA	
				195	Mammalian b FWD	GCG TAC GCA ATC TTA CGA TCA A	
					Mammalian b REV	CTG GCC TCC AAT TCA TGT GAG	
				395	Mammalian c FWD	CCA TCC AAC ATC TCA GCA TGA TGA AA	
					Mammalian c REV	GCC CCT CAG AAT GAT ATT TGT CCT CA	
mtDNA	Cyt b	Berdjane-Brouk et al. 2012 [19]	Vertebrates	350	cyt-AVZF	CCT CAG AAT GAT ATT TGT CCT C	PCR
					Cyt-AVZR	ATC CAA CAT CTC AGC ATT GAT GAA	
mtDNA	Cyt b	Tobe and Linacre 2008 [20]	Guinea pigs	94	Universal 2	TGA GGA CAA ATA TCA TTY TGA GGR GC	PCR
					C. porcellus 85	GAG GGA GCC GAA GTT TCA TCA CGT	
				156	Universal 1	GAC CAA TGA TAT GAA AAA CCA TCG TTG T	
					C. porcellus 471	CCA GAT TCA CTC TAC AAG GGT TGT C	
			Dogs	170	Universal 1	GAC CAA TGA TAT GAA AAA CCA TCG TTG T	
					C. lupus familiaris 99	CAA GCA TAC TCC TAG TAA GGA TCC G	
				303	Universal 2	TGA GGA CAA ATA TCA TTY TGA GGR GC	
					C. lupus familiaris 684	GAG TAG GAG TAA GGC TCC TAG GAT A	

DNA Target	Target Locus	Primer Source	Species Design	Size (nt)	Primer Name	Sequence	Assay Use
mtDNA	Cyt b	Tobe and Linacre 2008 [20]	Rabbits	184	Universal 1	GAC CAA TGA TAT GAA AAA CCA TCG TTG T	PCR
					O. cuniculus 115	GTG AAA ATT TGA ATT ATA AGG CAC AG	
				192	Universal 2	TGA GGA CAA ATA TCA TTY TGA GGR GC	
					O. cuniculus 571	AAA GAG GAG GTG AAT TAA GAC TAA AGT	
			Pigs	199	Universal 2	TGA GGA CAA ATA TCA TTY TGA GGR GC	
					S. scrofa 147	TCT GAT GTG TAA TGT ATT GCT AAG AAC	
				219	Universal 1	GAC CAA TGA TAT GAA AAA CCA TCG TTG T	
					S. scrofa 580	CGT GCA GGA ATA GGA GAT GTA CGG C	
			Goats	273	Universal 1	GAC CAA TGA TAT GAA AAA CCA TCG TTG T	
					C. hircus 198	GCC ATA ATT TAC ATC TCG ACA AAT GTG AGT T	
				313	Universal 2	TGA GGA CAA ATA TCA TTY TGA GGR GC	
					C. hircus 693	TTA GAA CAA GAA TTA GTA GCA TGG CG	
			Humans	246	Universal 2	TGA GGA CAA ATA TCA TTY TGA GGR GC	
					H. sapiens 208	TTC AGC CAT AAT TTA CGT CTC GAG T	
				277	Universal 1	GAC CAA TGA TAT GAA AAA CCA TCG TTG T	
					H. sapiens 624	ATC GGA ATG GGA GGT GAT TCC TAG G	
			Deer	188	Universal 2	TGA GGA CAA ATA TCA TTY TGA GGR GC	
					C. elaphus 210	CGA ATA ATT CAG CCA TAA TTG ACA TCT CGA	
				285	Universal 1	GAC CAA TGA TAT GAA AAA CCA TCG TTG T	
					C. elaphus 567	AGT AAG TGT ACT ATA GCG AGT GCT GCG	
			Mice	151	Universal 3	TTT TTT TTT TTT CGV TCH ATY CCH AAY AAA CTA GG	
					M. minutus 260	GAA GGA ATA AGC AGA TAA AAA ATA TGG ATG	
				340	Universal 1	GAC CAA TGA TAT GAA AAA CCA TCG TTG T	
					M. minutus 960	TAC AAG GAT TCA GTA AAG TGT TTG GGA G	

DNA Target	Target Locus	Primer Source	Species Design	Size (nt)	Primer Name	Sequence	Assay Use
mtDNA	Cyt b	Tobe and Linacre 2008 [20]	Mice	186	Universal 3	TTT TTT TTT TTT CGV TCH ATY CCH AAY AAA CTA GG	PCR
					M. musculus 746	GTA GTT GTC TGG GTC TCC TAG TAT ATC	
				362	Universal 2	TGA GGA CAA ATA TCA TTY TGA GGR GC	
					M. musculus 996	CTC CAA TTC AGG TTA AGA TAA GT	
			Cats	89	Universal 2	TGA GGA CAA ATA TCA TTY TGA GGR GC	
					F. catus 471	TTT CCC TCA GAT YCA TTC TAC TAG TTC AGT C	
				180	Universal 3	TTT TTT TTT TTT CGV TCH ATY CCH AAY AAA CTA GG	
					F. catus 990	GAT TCA TGT TAG GGT TAG GAG ATC C	
			Hedgehogs	120	Universal 2	TGA GGA CAA ATA TCA TTY TGA GGR GC	
					E. europaeus 499	GTT AGA GTA GCT TTG TCA ACT GAA AAT GA	
			Foxes	125	Universal 3	TTT TTT TTT TTT CGV TCH ATY CCH AAY AAA CTA GG	
					V. vulpes 572	GGA GAA ATA AGA GAT GAA CCA TCG CTA ATG	
				196	Universal 2	TGA GGA CAA ATA TCA TTY TGA GGR GC	
					V. vulpes 939	TAA GGG GYC GGA ATA TTA TCC CA	
			Badgers	173	Universal 3	TTT TTT TTT TTT CGV TCH ATY CCH AAY AAA CTA GG	
					M. meles 624	TTT GTC AGA ATT RGA GGG GAT WCC AGA G	
				241	Universal 2	TGA GGA CAA ATA TCA TTY TGA GGR GC	
					M. meles 985	GTT ARG GTG ARG AGG TCT GCA AC	
			Cows	93	Universal 3	TTT TTT TTT TTT CGV TCH ATY CCH AAY AAA CTA GG	
					B. taurus 666	TAA GAT GTC CTT AAT GGT ATA GTA G	

DNA Target	Target Locus	Primer Source	Species Design	Size (nt)	Primer Name	Sequence	Assay Use
mtDNA	Cyt b	Tobe and Linacre 2008 [20]	Cows	287	Universal 2	TGA GGA CAA ATA TCA TTY TGA GGR GC	PCR
					B. taurus 903	GTG TGT AGT AGG GGG ATT AGA GCA	
			Rats	192	Universal 3	TTT TTT TTT TTT CGV TCH ATY CCH AAY AAA CTA GG	
					R. norvegicus 682	GGA ATA ATA GTA ATA TAA ATA CAC CTA GGA GGT C	
				310	Universal 2	TGA GGA CAA ATA TCA TTY TGA GGR GC	
					R. norvegicus 999	GGT TGG CCT CCG ATT CAT GTT AAG ACT	
			Donkeys	198	Universal 3	TTT TTT TTT TTT CGV TCH ATY CCH AAY AAA CTA GG	
					E. asinus 704	AAT ACT AGG GTT AGT AGG AGT AGG ACT A	
				327	Universal 2	TGA GGA CAA ATA TCA TTY TGA GGR GC	
					E. asinus 1014	TTC TAC TGG TTG GCC ACC A	
			Horses	208	Universal 3	TTT TTT TTT TTT CGV TCH ATY CCH AAY AAA CTA GG	
					E. caballus 705	AGA ATA ATA CTA GAG TTA GTA GGA GCA AGA TC	
				333	Universal 2	TGA GGA CAA ATA TCA TTY TGA GGR GC	
					E. caballus 1023	TAC GTA TGG GTG TTC CAC TGG C	
			Sheep	98	Universal 3	TTT TTT TTT TTT CGV TCH ATY CCH AAY AAA CTA GG	
					O. aries 710	GGC GTG AAT AGT ACT AGT AGC ATG AGG ATG A	
				336	Universal 2	TGA GGA CAA ATA TCA TTY TGA GGR GC	
					O. aries 906	GCT TTG ATG TAT GGA GGA GGG GTA TAA TT	
mtDNA	Cyt b	Garros et al. 2011 [21]	Deer	188	Universal 2 [20]	TGA GGA CAA ATA TCA TTY TGA GGR GC	PCR
					Cervus	AGT AAG TGT ACT ATA GCG AGT GCT GCG	
				220	Universal 2 [20]	TGA GGA CAA ATA TCA TTY TGA GGR GC	
					Capreolus	TTG TCC GCG TTT GAT GGG ATT CCT ATC	

DNA Target	Target Locus	Primer Source	Species Design	Size (nt)	Primer Name	Sequence	Assay Use
mtDNA	Cyt b	Sales et al. 2015 [22]	Dogs	118	FWD	AGC GCC GTC TAA CAT CTC TG	qPCR
					REV	TGT GGC TGT GTC CGA TGT AT	
			Horses	103	FWD	CAG CCA GTG GAA CAC CCA TA	
					REV	TGT TTT CGA TGG TGC TTG CG	
			Cats	108	FWD	AGA ATG GAT CTG AGG GGG CT	
					REV	AGG TGT ACT GCT GCT AAG GC	
			Chickens	104	FWD	CAG CAG ACA CAT CCC TAG CC	
					REV	GAA GAA TGA GGC GCC GTT TG	
			Humans	104	FWD	AGG CGT CCT TGC CCT ATT AC	
					REV	GTG ATT GGC TTA GTG GGC G	
			Rats	109	FWD	GAA TTG GGG GCC AAC CAG TA	
					REV	TCA ATG ATT CCG GAG ATT GGT	
mtDNA	Ctrl. Reg.	Purdue et al. 2000 [23]	Vertebrates	685	H16501A	ATG GCC CTG TAG AAA GAA C	PCR
					L15926	TAC ACT GGT CTT GTA AAC C	
mtDNA	Ctrl. Reg.	Kocher et al. 1989 [10]	Vertebrates	1000	L15926	TCA AAG CTT ACA CCA GTC TTG TAA ACC	PCR
					L16007	CCC AAA GCT AAA ATT CTA A	
					H00651	TAA CTG CAG AAG GCT AGG ACC AAA CCT	
mtDNA	Ctrl. Reg.	Latch et al. 2008 [24]	Deer	730	Odh-dLoopF	GAG CAA CCA ATC TCC CTG AG	PCR
					Odh-dLoopR	GTG TGA GCA TGG GCT GAT TA	
mtDNA	ND2	Cicero & Johnson 2001 [3]	Avians	1108	L5204	GCT AAC AAA GCT ATC GGG CCC AT	PCR
					L5494	AAT GCA TGA TCC ACC GGC CAA TGA GA	
					H5578	CCT TGG AGT ACT TCT GGG AAT CAG A	
					L5809	GCC TTC TCA TCC ATC TCC CAC CTA GGA TGA AT	
					H6034	TTG GTT AGT TCT TGG ATA ATG AGT CA	
					H6312	CTT ATT TAA GGC TTT GAA GGC C	

DNA Target	Target Locus	Primer Source	Species Design	Size (nt)	Primer Name	Sequence	Assay Use
mtDNA	ND3	Cicero & Johnson 2001 [3]	Avians	588	L10701	CTC TAC ACA ACC ATC TAC TGA TGA GG	PCR
					H11289	GAT AGT ATT ATG CTT TCT AGG CA	
mtDNA	d-loop	Collini et al. 2015 [25]	Muroidea rodents	175-176	FWD	TCT GGT TCT TAC TTC AGG GC	PCR
					REV	TTC ATG CCT TGA CGG CTA TG	
			Soricidae shrews	136-137	FWD	TCA GCC CAT GCC GAC ACA T	
					REV	GCC CCC ATA GAG AAT AAG CC	
			Dogs	146-147	FWD	CCG CAA CGG CAC TAA CTC TA	
					REV	CCA TTG ACT GAA TAG CAC CTT G	
			Deer	168-169	FWD	CGA TGG ACT AAT GAC TAA TCA G	
					REV	TTA TGG GGA TGC TCA AGA TG	
rRNA	12S	Kitano et al. 2007 [26]	Vertebrates	215	L1085	CCA AAA CTG GGA TTA GAT ACC C	PCR
					H1259	GTT TGC TGA AGA TGG CGG TA	
rRNA	12S	Kocher et al. 1989 [10]	Vertebrates	386	L1091	AAA AAG CTT CAA ACT GGG ATT AGA TAC CCC ACT AT	PCR
					H1478	TGA CTG CAG AGG GTG ACG GGC GGT GTG T	
rRNA	12S	Humair et al. 2007 [27]	Vertebrates	145	12S-6F	CAA ACT GGG ATT AGA TAC C	PCR
					B-12S-9R	AGA ACA GGC TCC TCT AG	
rRNA	12S	Melton et al. 2007 [28]	Vertebrates	104	12SF	ACT GGG ATT AGA TAC CCC ACT ATG	PCR
					12SR	ATC GAT TAT AGA ACA GGC TCC TC	
rRNA	12S	Roca et al. 2004 [29]	Solenodons (rare insectivores)	500	12S3F	GGG ATT AGA TAC CCC ACT ATG C	PCR
					12S5R	TGC TTA CCA TGT TAC GAC TT	

DNA Target	Target Locus	Primer Source	Species Design	Size (nt)	Primer Name	Sequence	Assay Use
rRNA	12S	Wodecka et al. 2014 [30]	Vertebrates	520	532fl2s	CAA ACT GGG ATT AGA TAC	PCR
					1102rl2	TGC TTA CCT TGT TAC GAC	
				440	539fl2s	GGA TTA GAT ACC CCA CTA TGC	
					1015r12s	TGA GGA GGG TGA CGG GCG GT	
rRNA	12S	Karlsson & Holmlund 2007 [31]	Mammals	111	Partial 12S FWD	CCC CAC GGG AAA CAG CAG T	PCR
					Partial 12S REV	CGC GGT GGC TGG CAC GAA AT	
rRNA	12S	Collini et al. 2015 [25]	*Passeriformes*	155-156	FWD	ATC CAC GAT ATT ACC TGA CCA TT	PCR
					REV	TAC CCC ATT GCT TCC ATT CC	
			Caprinae	158	FWD	TAA ATC TCG TGC CAG CCA	
					REV	GTA GGG TTA CTT TCG TCA T	
rRNA	12S	Léger et al. 2015 [32]	Sheep	145	FWD	CCA GCC TTC CTG TTA ACT TTC AAT AGA CT	qPCR
					REV	TTT AGT CCT GTG TGA TTC GAA GGG CG	
			Chickens	145	FWD	CTC GCT AAT AAG ACA GGT CAA GGT A	
					REV	TAG GGG GTA TGA TCT CAC TTT ACT G	
rRNA	12S & 16S	Valinski et al. 2014 [33]	Vertebrates	500	12-16S F	ACA CCG CCC GTC ACC CTC C	PCR
					12-16S R	AAC CAG CTA TCA CCA GGC TCG	
rRNA	16S	Kitano et al. 2007 [26]	Vertebrates	244	L2513	GCC TGT TTA CCA AAA ACA TCA C	PCR
					H2714	CTC CAT AGG GTC TTC TCG TCT T	
rRNA	16S	Curler et al. 2015 [34]	Amphibians		Amphibian FWD	CTG TTT ACC AAA AAC ATC G	PCR
					Vertebrata REV	CTG ATC CAA CAT CGA GGT CGT	
rRNA	16S	Sawabe et al. 2010 [35]	Vertebrates	280	VerU-1	AAG ACG AGA AGA CCC YAT GGA	PCR
					VerU-2	CCT GAT CCA ACA TMG AGG TCG TA	

DNA Target	Target Locus	Primer Source	Species Design	Size (nt)	Primer Name	Sequence	Assay Use
rRNA	16S	Omondi et al. 2015 [36]	Vertebrates	200	Vert 16S For	GAG AAG ACC CTR TGG ARC TT	PCR
					Vert 16S Rev	CGC TGT TAT CCC TAG GGT A	
rRNA	16S	Karlsson & Holmlund 2007 [31]	Mammals	98	Partial 16S FWD	GAC GAG AAG ACC CTA TGG AGC	PCR
					Partial 16S REV	TCC GAG GTC GCC CCA ACC	
nuDNA	PNOC	Murphy et al. 2001 [37]	All eutherian orders (Human Chromosome #8)	333	PNOC FWD	GCA TCC TTG AGT GTG AAG AGA A	PCR
					PNOC REV	TGC CTC ATA AAC TCA CTG AAC C	
nuDNA	β2-Micro-globulin	Canavez et al. 1999 [38]	Primates	Var.	Exon 1 F1	GCT GGC TTG GAG ACC GGT GA	PCR
					Exon 1 F2	TTG GAG ACA GGT GAC GGT CCC	
					Exon 1 R1	AAA ACT TGG AGG GAG GTA ACG	
					Exon 1 R7	CAG GAA GGA AAA CTT GGA GG	
					Exon 2 F3	ATC CCC AAT TGA AAT ACC CTG	
					Exon 2 F8	GTG CCT AAT CTA GCT TGA GAC	
					Exon 2 R2	TTC AGC AGC CTA CAA AAG AA	
					Exon 2 R3	ACT TAC CCC ACT TTA ATG TCT T	
					Exon 2 R8	ATG ACA TGA CTA CCC ATA CAC A	
					Exon 3 F4	AAA AGT AAA ACT TAA TGT CTT CCT	
					Exon 3 F5	ATT TTT CTT TCT CCG CTG TCT	
					Exon 3 R4	CCT GTG ATA CCA CAC TGG CAG	
					Exon 3 R5	TAA CCA CCT GCC TCT ATC CTG	
					Exon 3 R9	TGT GGA AAT GGC AGA AGA AAG A	
					Intron 2 F7	CTC ACC ACC CAA GAC AGT AAA GT	
					Intron 2 F6	GAG TAT GCC TGC CGT GTG AG	

DNA Target	Target Locus	Primer Source	Species Design	Size (nt)	Primer Name	Sequence	Assay Use
nuDNA	β2-Micro-globulin	Canavez et al. 1999 [38]	Primates	Var.	Intron 2 R6	TGA AAA AGA CGA TGG AGA AAG AAA A	PCR
					Intron 2 R12	AGT GGA GAA AGA AAA AGG AAG A	
Trans-posable element	Alu	Walker et al. 2003 [39]	Primates	Var.	Alu 3	GAT CGC GCC ACT GCA CTC C	PCR
					Alu 8	GGA TTA CAG GCG TGA GCC AC	
					Intra-Yb8 48-69	CGA GGC GGG TGG ATC ATG AGG T	
					Intra-Yb8 273-254	TCT GTC GCC CAG GCC GGA CT	
					Intra-Yb6 FWD	GAG ATC GAG ACC AC/ GGT GAA A	
					Intra-Yb6 REV	TTT GAG ACG GAG TCT CGT T	

References

1. Ivanova, N.V.; Zemlak, T.S.; Hanner, R.H.; Herbert, P.D. Universal primer cocktails for fish DNA barcoding. *Mol. Ecol. Notes* **2007**, *7*, 5.
2. Townzen, J.S.; Brower, A.V.; Judd, D.D. Identification of mosquito bloodmeals using mitochondrial cytochrome oxidase subunit I and cytochrome b gene sequences. *Med. Vet. Entomol.* **2008**, *22*, 386-393.
3. Cicero, C.; Johnson, N.K. Higher-level phylogeny of new world vireos (aves: vireonidae) based on sequences of multiple mitochondrial DNA genes. *Mol. Phylogenet. Evol.* **2001**, *20*, 27-40.
4. Reeves, L.E.; Gillett-Kaufman, J.L.; Kawahara, A.Y.; Kaufman, P.E. Barcoding blood meals: New vertebrate-specific primer sets for assigning taxonomic identities to host DNA from mosquito blood meals. *PLoS Negl. Trop. Dis.* **2018**, *12*, e0006767.
5. Nagy, Z.T.; Sonet, G.; Glaw, F.; Vences, M. First large-scale DNA barcoding assessment of reptiles in the biodiversity hotspot of Madagascar, based on newly designed COI primers. *PloS one* **2012**, *7*, e34506.
6. Alcaide, M.; Rico, C.; Ruiz, S.; Soriguer, R.; Munoz, J.; Figuerola, J. Disentangling vector-borne transmission networks: a universal DNA barcoding method to identify vertebrate hosts from arthropod bloodmeals. *PloS one* **2009**, *4*, e7092.
7. Parodi, B.; Aresu, O.; Bini, D.; Lorenzini, R.; Schena, F.; Visconti, P.; Cesaro, M.; Ferrera, D.; Andreotti, V.; Ruzzon, T. Species identification and confirmation of human and animal cell lines: a PCR-based method. *Biotechniques* **2002**, *32*, 432-434, 436, 438-440.

8. Boakye, D.A.; Tang, J.; Truc, P.; Merriweather, A.; Unnasch, T.R. Identification of bloodmeals in haematophagous *Diptera* by cytochrome B heteroduplex analysis. *Med. Vet. Entomol.* **1999**, *13*, 282-287.
9. Meece, J.K.; Reynolds, C.E.; Stockwell, P.J.; Jenson, T.A.; Christensen, J.E.; Reed, K.D. Identification of mosquito bloodmeal source by terminal restriction fragment length polymorphism profile analysis of the cytochrome B gene. *J. Med. Entomol.* **2005**, *42*, 657-667.
10. Kocher, T.D.; Thomas, W.K.; Meyer, A.; Edwards, S.V.; Paabo, S.; Villablanca, F.X.; Wilson, A.C. Dynamics of mitochondrial DNA evolution in animals: amplification and sequencing with conserved primers. *Proc. Natl. Acad. Sci. U.S.A.* **1989**, *86*, 6196-6200.
11. Latch, E.K.; Heffelfinger, J.R.; Fike, J.A.; Rhodes, O.E., Jr. Species-wide phylogeography of North American mule deer (*Odocoileus hemionus*): cryptic glacial refugia and postglacial recolonization. *Mol. Ecol.* **2009**, *18*, 1730-1745.
12. Abbasi, I.; Cunio, R.; Warburg, A. Identification of blood meals imbibed by phlebotomine sand flies using cytochrome b PCR and reverse line blotting. *Vector Borne Zoonotic Dis.* **2009**, *9*, 79-86.
13. Mota, J.; Chacon, J.C.; Gutierrez-Cabrera, A.E.; Sanchez-Cordero, V.; Wirtz, R.A.; Ordonez, R.; Panzera, F.; Ramsey, J.M. Identification of blood meal source and infection with *Trypanosoma cruzi* of Chagas disease vectors using a multiplex cytochrome b polymerase chain reaction assay. *Vector Borne Zoonotic Dis.* **2007**, *7*, 617-627.
14. Ngo, K.A.; Kramer, L.D. Identification of mosquito bloodmeals using polymerase chain reaction (PCR) with order-specific primers. *J. Med. Entomol.* **2003**, *40*, 215-222.
15. Kent, R.J.; Norris, D.E. Identification of mammalian blood meals in mosquitoes by a multiplexed polymerase chain reaction targeting cytochrome B. *Am. J. Trop. Med. Hyg.* **2005**, *73*, 336-342.
16. Fornadel, C.M.; Norris, D.E. Increased endophily by the malaria vector Anopheles arabiensis in southern Zambia and identification of digested blood meals. *The American journal of tropical medicine and hygiene* **2008**, *79*, 876-880.
17. Molaei, G.; Andreadis, T.G.; Armstrong, P.M.; Anderson, J.F.; Vossbrinck, C.R. Host feeding patterns of *Culex* mosquitoes and West Nile virus transmission, northeastern United States. *Emerg. Infect. Dis.* **2006**, *12*, 468-474.
18. Sorenson, M.D.; Ast, J.C.; Dimcheff, D.E.; Yuri, T.; Mindell, D.P. Primers for a PCR-based approach to mitochondrial genome sequencing in birds and other vertebrates. *Mol. Phylogenet. Evol.* **1999**, *12*, 105-114.
19. Berdjane-Brouk, Z.; Kone, A.K.; Djimde, A.A.; Charrel, R.N.; Ravel, C.; Delaunay, P.; del Giudice, P.; Diarra, A.Z.; Doumbo, S.; Goita, S., et al. First detection of *Leishmania major* DNA in *Sergentomyia (Spelaeomyia) darlingi* from cutaneous leishmaniasis foci in Mali. *PloS one* **2012**, *7*, e28266.
20. Tobe, S.S.; Linacre, A.M. A multiplex assay to identify 18 European mammal species from mixtures using the mitochondrial cytochrome b gene. *Electrophoresis* **2008**, *29*, 340-347.

21. Garros, C.; Gardes, L.; Allene, X.; Rakotoarivony, I.; Viennet, E.; Rossi, S.; Balenghien, T. Adaptation of a species-specific multiplex PCR assay for the identification of blood meal source in *Culicoides* (Ceratopogonidae: Diptera): applications on Palaearctic biting midge species, vectors of Orbiviruses. *Infect. Genet. Evol.* **2011**, *11*, 1103-1110.
22. Sales, K.G.; Costa, P.L.; de Morais, R.C.; Otranto, D.; Brandao-Filho, S.P.; Cavalcanti Mde, P.; Dantas-Torres, F. Identification of phlebotomine sand fly blood meals by real-time PCR. *Parasit. Vectors* **2015**, *8*, 230.
23. Purdue, J.R.; Smith, M.H.; Patton, J.C. Female philopatry and extreme spatial genetic heterogeneity in white-tailed deer. *J. Mammal.* **2000**, *81*, 179-185.
24. Latch, E.K.; Amann, R.P.; Jacobson, J.P.; Rhodes, O.E. Competing hypotheses for the etiology of cryptorchidism in Sitka black-tailed deer: an evaluation of evolutionary alternatives. *Anim. Conserv.* **2008**, *11*, 234-246.
25. Collini, M.; Albonico, F.; Hauffe, H.C.; Mortarino, M. Identifying the last bloodmeal of questing sheep tick nymphs (*Ixodes ricinus* L.) using high resolution melting analysis. *Vet. Parasitol.* **2015**, *210*, 194-205.
26. Kitano, T.; Umetsu, K.; Tian, W.; Osawa, M. Two universal primer sets for species identification among vertebrates. *Int. J. Legal Med.* **2007**, *121*, 423-427.
27. Humair, P.F.; Douet, V.; Cadenas, F.M.; Schouls, L.M.; Van de Pol, I.; Gern, L. Molecular identification of bloodmeal source in *Ixodes ricinus* ticks using 12S rDNA as a genetic marker. *J. Med. Entomol.* **2007**, *44*, 869-880.
28. Melton, T.; Holland, C. Routine forensic use of the mitochondrial 12S ribosomal RNA gene for species identification. *J. Forensic. Sci.* **2007**, *52*, 1305-1307.
29. Roca, A.L.; Bar-Gal, G.K.; Eizirik, E.; Helgen, K.M.; Maria, R.; Springer, M.S.; O'Brien, S.J.; Murphy, W.J. Mesozoic origin for West Indian insectivores. *Nature* **2004**, *429*, 649-651.
30. Wodecka, B.; Rymaszewska, A.; Skotarczak, B. Host and pathogen DNA identification in blood meals of nymphal *Ixodes ricinus* ticks from forest parks and rural forests of Poland. *Exp. Appl. Acarol.* **2014**, *62*, 543-555.
31. Karlsson, A.O.; Holmlund, G. Identification of mammal species using species-specific DNA pyrosequencing. *Forensic Sci. Int.* **2007**, *173*, 16-20.
32. Leger, E.; Liu, X.; Masseglia, S.; Noel, V.; Vourc'h, G.; Bonnet, S.; McCoy, K.D. Reliability of molecular host-identification methods for ticks: an experimental in vitro study with *Ixodes ricinus*. *Parasit. Vectors* **2015**, *8*, 433.
33. Valinsky, L.; Ettinger, G.; Bar-Gal, G.K.; Orshan, L. Molecular identification of bloodmeals from sand flies and mosquitoes collected in Israel. *J. Med. Entomol.* **2014**, *51*, 678-685.
34. Curler, G.R.; Moulton, J.K.; Madriz, R.I. Redescription of *Aposycorax chilensis* (Tonnoir) (Diptera, Psychodidae, Sycoracinae) with the first identification of a blood meal host for the species. *Zootaxa* **2015**, *4048*, 114-126.
35. Sawabe, K.; Isawa, H.; Hoshino, K.; Sasaki, T.; Roychoudhury, S.; Higa, Y.; Kasai, S.; Tsuda, Y.; Nishiumi, I.; Hisai, N., et al. Host-feeding habits of *Culex pipiens* and *Aedes albopictus* (Diptera: Culicidae) collected at the urban and suburban residential areas of Japan. *J. Med. Entomol.* **2010**, *47*, 442-450.

36. Omondi, D.; Masiga, D.K.; Ajamma, Y.U.; Fielding, B.C.; Njoroge, L.; Villinger, J. Unraveling host-vector-arbovirus interactions by two-gene high resolution melting mosquito bloodmeal analysis in a Kenyan wildlife-livestock interface. *PloS one* **2015**, *10*, e0134375.
37. Murphy, W.J.; Eizirik, E.; Johnson, W.E.; Zhang, Y.P.; Ryder, O.A.; O'Brien, S.J. Molecular phylogenetics and the origins of placental mammals. *Nature* **2001**, *409*, 614-618.
38. Canavez, F.C.; Moreira, M.A.M.; Ladasky, J.J.; Pissinatti, A.; Parham, P.; Seuanez, H.N. Molecular Phylogeny of NewWorld Primates (Platyrrhini) Based on b2-Microglobulin DNA Sequences. *Mol. Phylogenet. Evol.* **1999**, *12*, 74-82.
39. Walker, J.A.; Kilroy, G.E.; Xing, J.; Shewale, J.; Sinha, S.K.; Batzer, M.A. Human DNA quantitation using Alu element-based polymerase chain reaction. *Anal. Biochem.* **2003**, *315*, 122-128.

Publisher's Note: MDPI stays neutral with regard to jurisdictional claims in published maps and institutional affiliations.

© 2021 by the authors. Submitted for possible open access publication under the terms and conditions of the Creative Commons Attribution (CC BY) license (http://creativecommons.org/licenses/by/4.0/).
